# Functional Comparison of Horizontal Mattress Suture *Versus* Free‐Edge Suture in the All‐Inside Arthroscopic Broström–Gould Procedure for Chronic Lateral Ankle Instability

**DOI:** 10.1111/os.12817

**Published:** 2020-10-18

**Authors:** Shi‐Ming Feng, Meng Han, Ai‐Guo Wang, Jia‐Qiang Fan

**Affiliations:** ^1^ Orthopaedic Department, Sports Medicine Department Xuzhou Central Hospital Xuzhou China; ^2^ Orthopaedic Department Xuzhou Clinical College of Xuzhou Medical University Xuzhou China

**Keywords:** Broström–Gould procedure, Chronic lateral ankle instability, Free‐edge suture, Horizontal mattress suture

## Abstract

**Objective:**

To compare the clinical outcomes of horizontal mattress suture *vs* free‐edge suture in the all‐inside arthroscopic Broström–Gould procedure.

**Methods:**

This retrospective cohort study included 68 chronic lateral ankle instability (CLAI) patients who underwent either a horizontal mattress suture or a free‐edge suture all‐inside arthroscopic Broström–Gould procedure from January 2014 to January 2017. Patients were divided into two groups based on the suture fashion during the all‐inside arthroscopic Broström–Gould procedure. In the horizontal mattress suture group (n = 31), anchor sutures were used to suture the ATFL, capsule, and inferior extensor retinaculum in horizontal mattress suture fashion. In the free‐edge suture group (n = 37), anchor sutures were used to suture the ATFL, capsule, and inferior extensor retinaculum in free‐edge suture fashion. The Visual Analogue Scale (VAS) score, the American Orthopaedic Foot and Ankle Society (AOFAS) score, Karlsson Ankle Functional Score (KAFS), Anterior Talar Translation (ATT), the rate of return to sports, and ankle proprioceptive recovery were compared in both groups.

**Results:**

The operative times and duration of hospitalization between the two groups were comparable (all *P* > 0.05). The VAS, AOFAS, ATT, the rate of return to sports, and ankle proprioceptive recovery were comparable between the horizontal mattress suture and free‐edge suture groups at 1 and 2 years after surgery. Patients of the free‐edge suture group achieved better KAFS 1 and 2 years after the surgery compared with those of the horizontal mattress suture group. In both groups, incisions were healed by first intention, and complications such as infection, implant reactions, tendon injury, and nervous or vascular injuries were not observed. The ankle proprioceptive recovery in horizontal mattress suture and free‐edge suture groups showed no significant differences at 1 and 2 years after surgery. The mean time of the return to full activity for patients in the horizontal mattress suture group was 10.38 ± 2.02 (range 8 to 12) weeks *vs* 8.63 ± 2.31 (range 8 to 12) weeks for those in the free‐edge suture group (*P* = 0.001, power = 0.907). The exercise participation rates were comparable between groups (*P* > 0.05). At the 2‐year follow‐up, all patients regained normal activities and ankle stability, and no recurrence of CLAI or revision surgery was recorded.

**Conclusion:**

All‐inside arthroscopic Broström–Gould surgery for the treatment of CLAI ensures a better functional effect (KAFS) and better recovery time when free‐edge suture is used instead of horizontal mattress suture.

## Graphical abstract

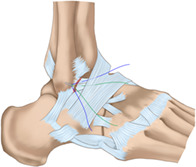

All‐inside arthroscopic Broström–Gould surgery for chronic lateral ankle instability with free‐edge suture provides better functional results compared with horizontal mattress suture.

## Introduction

The Broström–Gould procedure is currently the first choice for the treatment of chronic lateral ankle instability (CLAI); good‐to‐excellent results have been reported^1^, ^2^. The procedure allows immediate weight‐bearing and returns high‐demand athletes to their preinjury levels. The classical Broström–Gould procedure is composed of the following steps: repairing the lateral ligaments, tightening the joint capsule, and strengthening the inferior extensor retinaculum^3^. For the open Broström–Gould procedure, numerous reports addressed the ligament suture fashion of the lateral ligament complex, and the mostly used fashion was the pants‐over‐vest, with the anterior talofibular ligament (ATFL) and the inferior extensor retinaculum re‐sutured. Maffulli *et al*.^4^ used a vest‐over‐pant suture fashion to repair the ATFL in 42 patients, and 38 patients were followed up for 8.7 years (range, 5–13 years). Significant improvement of ankle stability, American Orthopaedic Foot and Ankle Society (AOFAS) scores, and Kaikkonen scales were observed. The mean AOFAS and Kaikkonen score improved from 51 to 90, 45 to 90, respectively. The anterior drawer test showed a significant improvement compared with pre‐surgery, with grade 0 in 19 patients, grade 1 in 11 patients, and grade 2 in eight patients.

Intra‐articular lesions in the CLAI cases are the strongest indicators of poor clinical outcomes. Functional outcomes are poor if intra‐articular lesions are not properly treated^5^. With recent development of arthroscopic instruments and surgical techniques, the arthroscopic Broström–Gould procedure has been widely used in recent years^6^, ^7^. The arthroscopic procedure not only repairs the lateral ligament and strengthens the inferior extensor retinaculum, but also simultaneously treats the intra‐articular lesions, achieving the same fixation strength and functional effect as the open Broström–Gould procedure^8^, ^9^. Among the investigations dealing with the functional outcomes of the open surgery *vs* arthroscopic surgery, Rigby and collaborators^10^ treated 32 patients with open Broström–Gould procedure and 30 patients with all‐inside Bröstrom procedure. The functional or patient satisfaction outcome scores were comparable for the two groups. Woo and coworkers^11^ retrospectively reviewed and compared outcomes of 52 CLAI patients treated with the Broström–Gould procedure utilizing open surgery (26 patients) and arthroscopic surgery (26 patients). After a follow‐up of 12 months, the arthroscopic group demonstrated significantly higher AOFAS scores. With the proved advantages of minimally invasive and faster recovery, arthroscopic surgery offers an alternative procedure to open surgery.

However, compared to the open Broström–Gould procedure, the lateral ligament complex was plicated instead of imbricated during the arthroscopic Broström–Gould procedure. Feng *et al*.^12^ used the all‐inside arthroscopic Broström–Gould procedure for 75 CLAI patients. The patients were divided into a single‐anchor group (n = 36) and double‐anchor group (n = 39) according to the number of anchors used. The ATFL and inferior extensor retinaculum were plicated during the operation. Improvement of Visual Analogue Scale (VAS), AOFAS, Karlsson Ankle Functional Score (KAFS), and Foot and Ankle Outcome Score in both groups were observed. Cho and coworkers^13^ treated 22 patients with medial gutter osteoarthritis related to CLAI by using the modified Broström procedure and arthroscopic debridement. The ATFL and inferior extensor retinaculum were imbricated. At the final follow‐up, the mean AOFAS scores improved from 51.2 points (range, 38–67 points) to 80.3 points (range, 58–95 points); the mean VAS scores significantly decreased from 6.8 points (range, 4–9 points) to 3.5 points (range, 0–8 points). Yeo and coworkers^9^ prospectively analyzed and compared outcomes of 50 CLAI cases treated with modified Broström operation utilizing all‐inside arthroscopic procedure (25 patients) and open procedure (25 patients). The ATFL and inferior extensor retinaculum were sutured in pants‐over‐vest fashion in the open group, and plication fashion in the arthroscopic group. After a follow‐up at 12 months, both groups had the same functional outcomes (KAFS AOFAS, VAS, Anterior Talar Translation [ATT], and talar tilt).

The above literature indicated that, when considering the Broström–Gould procedure for CLAI, suturing the ATFL and inferior extensor retinaculum in pants‐over‐vest fashion or plication fashion could reach satisfied functional outcomes. All the above suture configurations produced satisfactory ankle functional results. The current literature reports mainly focus on the number of the anchors instead of the anchor suture fashion, as the suture anchor is the most used fixator for Broström–Gould procedure^14^, ^15^. Numerous reports addressed differences in biomechanical and clinical outcomes between the use of one *vs* two anchors^12^, ^16^, ^17^. Nevertheless, there are no reports addressing the differences in clinical outcomes between suture techniques in the arthroscopic Broström–Gould procedure.

Therefore, the purpose of this present retrospective study was as follows. First, we aimed to investigate the clinical outcomes of arthroscopic Broström–Gould surgery in the treatment of CLAI through a follow‐up of 2 years. Second, we aimed to compare the functional results of all‐inside arthroscopic Broström–Gould surgery for CLAI with horizontal mattress suture and free‐edge suture over 2 years of follow‐up. Third, we analyzed the complications, such as infection, nerve and tendon injury, and rejection. And we used VAS, AOFAS, KAFS, ATT, the rate of return to sports, and Active Joint Position Sense (AJPS) criteria in an effort to provide evidence‐based recommendations regarding the suture technique to be used in Broström–Gould procedure.

## Materials and Methods

This was a retrospective cohort study evaluating the clinical results of the all‐inside arthroscopic Broström–Gould procedures with horizontal mattress suture and free‐edge suture used to treat CLAI. The institutional review boards of our hospital approved the study. All patients provided signed informed consent as well as consent under the Health Insurance Portability and Accountability Act to participate in this study.

### *Patient Selection*

Inclusion criteria were: (i) CLAI patients unresponsive to a minimum of 6 months of appropriately conducted conservative management, including rest, bracing, use of anti‐inflammatory drugs, proprioceptive and balance training, strengthening of the peroneal muscles, and physical therapy; (ii) received unilateral all‐inside arthroscopic Broström–Gould procedure with one suture anchor fixation (Fastin RC 3.5 mm, Smith & Nephew, Andover, MA) from January 2014 to January 2017; (iii) the suture arms of the anchor sutured in free‐edge suture fashion were considered as the comparison; (iv) complete surgical data and follow‐up outcomes and follow‐up time was not less than 24 months; (v) the study was designed as a retrospective cohort study.

Exclusion criteria were: (i) combined foot and ankle deformity, abnormal lower limb alignment, fracture, ankylosis, and other ligament injuries; (ii) combined central and peripheral neuromuscular disorders or ligamentous laxity; (iii) ankle osteoarthritis or other lesions in the joint (osteochondral lesions, impingement syndrome, Os subfibulare, sinus tarsi syndrome); (iv) previous ankle injury or surgery on the affected ankle, or secondary ankle injury during the follow‐up period.

### *Participants*

During the 37‐month period of the study, 185 consecutive CLAI patients underwent the all‐inside arthroscopic Broström–Gould procedure by a senior surgeon with extensive experience in foot and ankle surgery. Of these, 22 patients were lost to follow‐up, and 30 patients were followed for less than 24 months. Nineteen patients had osteochondral lesions, 11 patients had sinus tarsi syndrome, and 13 patients had ankle osteoarthritis. Eight patients had received previous surgery of the affected ankle and 14 patients underwent the procedure with two anchor fixations.

**Fig. 1:**
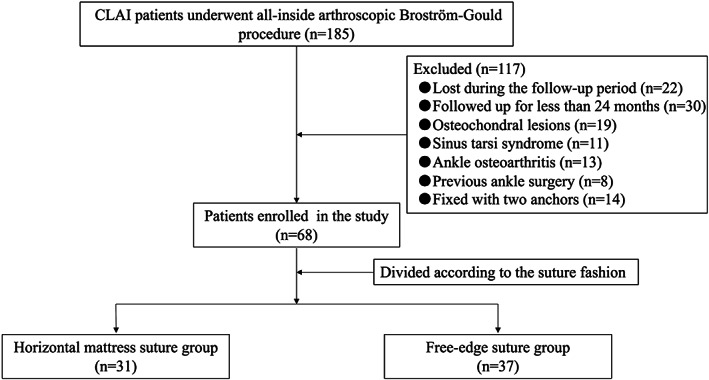
The flow diagram of the study. CLAI, chronic lateral ankle instability.

After all exclusions, the study included 68 patients. Patients were divided into two groups based on the suture fashion during the all‐inside arthroscopic Broström–Gould procedure. In the horizontal mattress suture group (n = 31), anchor sutures were used to suture the ATFL, capsule, and inferior extensor retinaculum in horizontal mattress suture fashion. In the free‐edge suture group (n = 37), anchor sutures were used to suture the ATFL, capsule, and inferior extensor retinaculum in free‐edge suture fashion (Fig. 1).

There was no statistical difference in the general preoperative data between the two groups (Table 1); in addition, differences in VAS score, the AOFAS score, KAFS, and ATT between the two groups were not statistically significant (Table 2).

**TABLE 1:** General information of the patients of two groups

Variable	Horizontal mattress suture group (n = 31)	Free‐edge suture group (n = 37)	*P* * value
Age, y	28.63 ± 11.21	30.37 ± 9.18	0.492^‡^
Sex			0.941^†^
Male	19	23	
Female	12	14	
Body Mass Index, kg/m^2^	21.84 ± 3.37	23.05 ± 2.53	0.105^‡^
Disease duration, mo	13.90 ± 6.82	14.55 ± 5.48	0.671^‡^

* A value *P* < 0.05 was set as statistically significant

^†^ Pearson χ^2^ test

^‡^ *t* test.

**TABLE 2:** Comparison of preoperative assessment outcomes in two groups of patients

Variable	Horizontal mattress suture group (n = 31)	Free‐edge suture group (n = 37)	*P* * value
VAS	6.17 ± 2.84	6.63 ± 3.20	0.532^†^
AOFAS	70.72 ± 10.33	68.97 ± 9.86	0.480^†^
KAFS	64.11 ± 9.67‐	67.38 ± 9.05	0.158^†^
ATT, mm	10.75 ± 4.72	11.26 ± 3.28	0.614^†^

AOFAS, American Orthopedic Foot and Ankle Society; ATT, Anterior Talar Translation; KAFS, Karlsson Ankle Functional Score; VAS, Visual analogue scale.

* A value *P* < 0.05 was set as statistically significant

^†^ t test.

### *Surgical Technique*

#### *Anesthesia and Position*

With the patient supine, a 7‐cm cushion was placed under the affected hip after induction of spinal or general anesthesia. The affected leg was placed over the distal edge of the operating table for convenient operation technique. A pressure pneumatic tourniquet was placed at the middle segment of the thigh, and inflated to 60 kPa after exsanguination.

#### *Approach, Exposure, and Arthroscopic Debridement*

Standard anterolateral and anteromedial ankle portals were established. Any identified intra‐articular pathology was fully evaluated and addressed. The accessory anterior portal to the fibular apex was made to better evaluate and manage the ATFL. The proliferative surrounding tissue, the synovial tissue, and periosteum were shaved distal to ATFL. The footprint region on the anterior side of the distal fibula was exposed and a bleeding bony surface was created using a motorized burr.

#### *Anchor Insertion*

A suture anchor (Fastin RC 3.5 mm, Smith & Nephew, Andover, MA) was inserted into the mid‐portion of the footprint area of the fibula through the accessory anterior portal.

#### *Repair the Ligament*

**Fig. 2:**
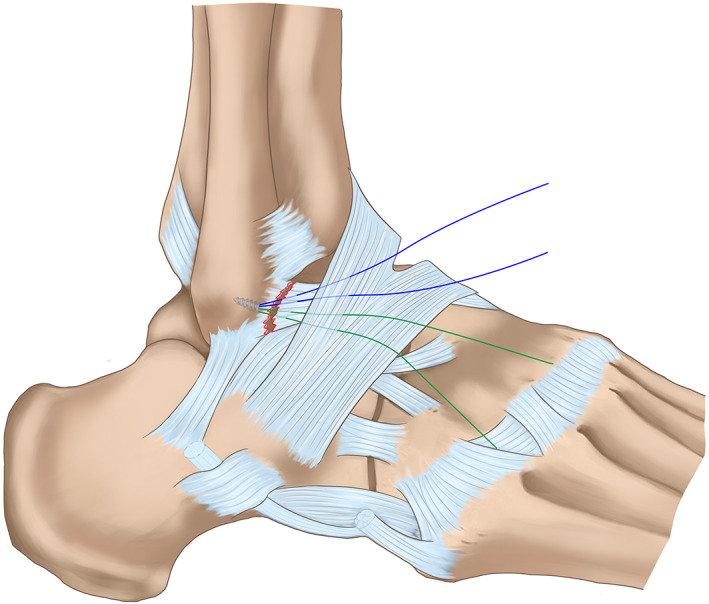
Surgical diagrams of arthroscopic Broström–Gould procedure with horizontal mattress suture fashion.

**Fig. 3:**
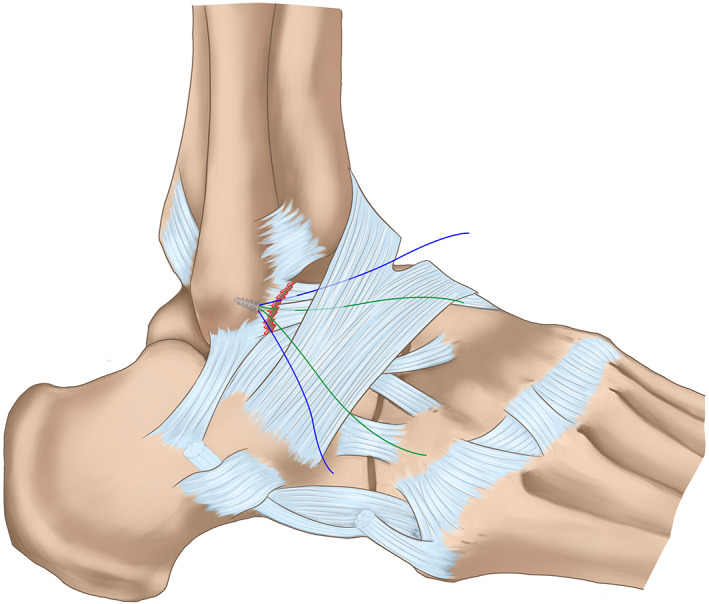
Surgical diagrams of arthroscopic Broström–Gould procedure with free‐edge suture fashion.

**Fig. 4:**
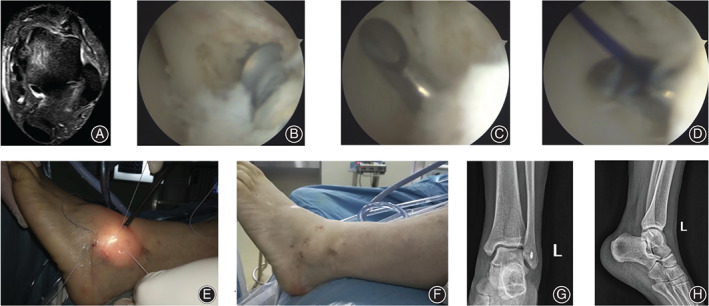
A 45‐year‐old male patient with chronic lateral ankle instability of the left side for 12 months. (A) The preoperative MRI showed the interruption of the ATFL. (B) The intra‐operative view under the arthroscope showed the ATFL interruption, a suture anchor was fixed into the footprint of the distal fibular. (C) A suture passer was used to pass through the ATFL. (D) The PDS II was passed through the suture passer and guided the suture arm to pass through ATFL and the inferior extensor retinaculum. (E) The arms of the suture anchor were knotted by free‐edge suture fashion. (F) The surgical approaches view after the procedure. (G) The postoperative anterior–posterior X‐ray film of the involved ankle. (H) The lateral X‐ray film after the surgery.

**Fig. 5:**
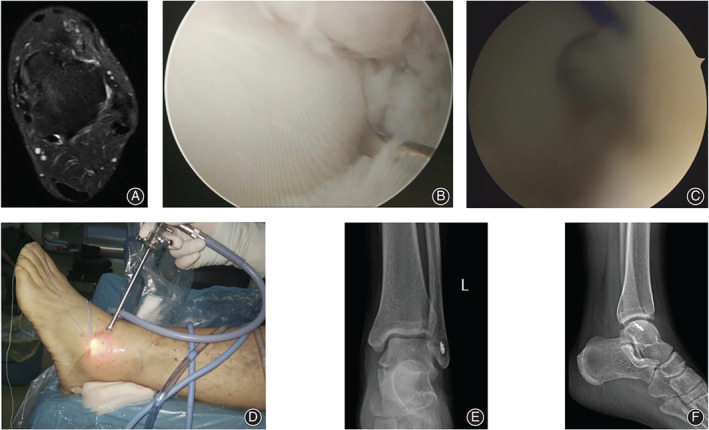
A 28‐year‐old male patient with chronic lateral ankle instability for 15 months after failure of 8 months of conservative treatment. (A) The preoperative MRI showed the integrity of the ATFL was interrupted. (B) The intra‐operative view under the arthroscope showed the ATFL detachment at the fibular side, and the laxity of the ATFL was recorded after probe palpation. (C) A suture passer was used to pass the anchor arms through the ATFL and inferior extensor retinaculum. (D) The anchor arms were sutured by horizontal mattress suture fashion. (E) The postoperative anterior–posterior X‐ray film of the operated ankle. (F) The lateral X‐ray film of the left ankle after the surgery.

**Fig. 6:**
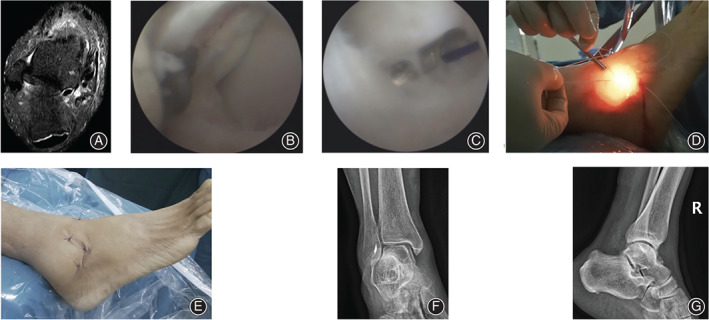
A 40‐year‐old female patient with chronic lateral ankle instability. (A) The preoperative MRI showed the integrity of the ATFL was interrupted of the right ankle. (B) The intra‐operative view under the arthroscope showed a suture anchor was introduced into the distal fibular. (C) A suture passer with PDS II was used to pass through the ATFL and inferior extensor retinaculum. (D) The anchor arms were sutured by free‐edge suture fashion. (E) The postoperative appearance of the portals. (F) The anterior–posterior X‐ray film of the right ankle after surgery. (G) The postoperative lateral X‐ray film of the involved ankle.

In the horizontal mattress suture group, the ATFL, capsule, and inferior extensor retinaculum were augmented with horizontal mattress sutures (Fig. 2). In the free‐edge suture group, the ATFL, capsule, and inferior extensor retinaculum were sutured together in turns with free‐edge sutures (Fig. 3). With the foot everted and dorsiflexed, the suture knot was tightened with a knot pusher (typical cases are shown in Figs 4, 5, 6).

### *Postoperative Management*

A short leg cast was used to immobilize the operated ankle for 2 weeks in slight dorsiflexion and eversion without weightbearing. On the second day after surgery, the patient was advised to perform early non‐weightbearing functional exercises and isometric exercises of lower limb muscles. Then, a short leg walking cast was used in the next 2 weeks, and progressive weightbearing was allowed. The Aircast™ (DJO, Vista, CA, USA) was used in the next 4 weeks, with fully weight‐bearing functional exercises. At approximately 8 weeks postoperatively, after removing the Aircast, patients were instructed to begin running and functional activities.

### *Postoperative Follow‐Up and Observational Indexes*

Wound healing and ankle stability were assessed after surgery; VAS, AOFAS, KAFS, ATT, were administered and time of return to sports and rate of return to pre‐injury sports was measured to assess ankle function and proprioception. Ankle proprioception was assessed using the AJPS. All measurements were undertaken by the same rehabilitation physician who did not participate in surgery and was blind to the procedure.

### *Outcome Evaluation*

All the patients received the guidance of a professional physician before scoring, in order to better record the ankle function of patients.

#### *Visual Analogue Scale (VAS)*

VAS is the most commonly used score system for pain. A 10‐point VAS was used for assessment, with a rating of 0 for signs of no pain, and 10 for sings of intense pain. The higher the score, the greater the pain intensity. The following cut points was used: mild pain (0–3 points), moderate pain (4–6 points), and severe pain (7–10 points).

#### *American Orthopaedic Foot & Ankle Society Scale (AOFAS)*

AOFAS score system has been widely used as a region‐specific health outcome measure to assess foot and ankle outcomes. The AOFAS scale is a 100‐point score system with three categories: pain (40 points), function (50 points), and alignment (10 points). A total score <50 is considered a poor result, 50–74 is fair, 75–89 is good, and 90–100 is considered an excellent outcome.

#### *Karlsson Ankle Functional Score (KAFS)*

Karsson ankle function score system is the most widely used activity scoring system for patients after ankle surgery. The KAFS scale ranges from 0 to 100 points and is based on the following eight items: pain, swelling, subjective instability, stiffness, stair climbing, running, work and life, and the use of ankle support aids. A higher score represents a higher level of ankle function.

#### *Anterior Talar Translation (ATT)*

ATT was measured in the anterior drawer stress radiographs using a spring dynamometer with a loading force of 150 N. The test was repeated three times by two surgeons, and the averaged outcomes were recorded.

#### *Active Joint Position Sense (AJPS)*

AJPS was measured using the active joint angle reproduction test. The patients were seated on a height‐adjustable table with the affected foot placed at a 90° angle from the hip, knee, and ankle. The affected ankle was passively placed in 10° and 20° of inversion and plantar flexion, respectively, three times, using the footplate. The patients were then asked to actively place the foot in these positions.

#### *Complications*

The evaluation of surgical complications is of great significance to the feasibility and safety of the operation. All the following postoperative complications were evaluated and recorded by three experienced foot and ankle surgeons that were totally unaware of the operation and procedures: infection, nerve injury, blood vessel injury, tendon injury, implant rejection, ankle pain, lateral ankle stability, and instability recurrence. If there were three different opinions, a final conclusion was made after discussion.

### *Statistical Analysis*

The SPSS 17.0 software (SPSS, Inc., Chicago, IL, USA) was used for analysis. The quantitative variables were expressed as mean ± standard deviation. The measurement data (VAS, AOFAS, FAOS, ATT, and AJPS scores) before and after surgery and between the two groups after surgery were compared using the Student's t‐test (for normal distribution) or the Mann–Whitney test (for asymmetric distribution). The Pearson chi‐square test was used to compare categorical variables. The univariate analysis was used to analyze the correlation of continuous variables. The Spearman test was used to evaluate associations among functional outcomes of age, body mass index (BMI), and VAS. The α value was set as 0.05 due to the univariate comparisons before and after surgery. A *post hoc* power analysis was performed. A *P*‐value < 0.05 was considered statistically significant.

## Results

### *Follow‐Up*

The patients were followed up from the time they were discharged. The functional outcomes and complications were recorded at the follow‐up time. The follow‐up time points and data were collected at 1 and 2 years after the operation.

### *General Results*

All patients underwent ankle synovial tissue debridement. During the operation, 13 patients underwent arthroscopic microfracture of the talus. In the horizontal mattress suture group, the operative time ranged from 30 to 80 min, with an average of 48.18 ± 10.37 min. In the free‐edge suture group, the operative time ranged from 25 to 75 min, with an average of 45.86 ± 8.44 min. The duration of hospitalization ranged from 3 to 5 days, with an average of 3.85 ± 1.62 days and 3.77 ± 1.19 days in the horizontal mattress suture and free‐edge suture groups, respectively. The operative times and duration of hospitalization between the groups were comparable (Table 3, t = 0.999, *P* = 0.322, power = 0.166; *t*= 0.228, *P* = 0.820, power = 0.056).

**TABLE 3:** Comparison of surgical characteristics and postoperative clinical outcomes between the two groups

Variable	Horizontal mattress suture group (n = 31)	Free‐edge suture group (n = 37)	*P* value*	Power^‡^
OT, min	48.18 ± 10.37	45.86 ± 8.44	0.322^†^	0.166
LHS, day	3.85 ± 1.62	3.77 ± 1.19	0.820^†^	0.056
VAS				
1 year	1.42 ± 0.76	1.57 ± 0.93	0.467^†^	0.111
2 years	1.03 ± 1.10	1.09 ± 0.88	0.807^†^	0.057
AOFAS				
1 year	90.36 ± 8.52	91.24 ± 9.21	0.684^†^	0.069
2 years	93.17 ± 5.83	93.85 ± 6.46	0.650^†^	0.073
KAFS				
1 year	82.51 ± 7.02	87.16 ± 6.14	0.022^†^	0.809
2 years	86.26 ± 6.18	90.54 ± 5.23	0.003^†^	0.850
ATT, mm				
1 year	3.81 ± 1.57	3.93 ± 1.63	0.759^†^	0.061
2 years	3.34 ± 1.22	3.40 ± 1.45	0.854^†^	0.054

AOFAS, American Orthopaedic Foot and Ankle Society; ATT, Anterior Talar Translation; KAFS, Karlsson Ankle Functional Score; LHS, Length of hospital stay; OT, Operative time; VAS, Visual analogue scale.

* A value *P* < 0.05 was set as statistically significant

^†^ *t* test

^‡^ Power is computed to reject the null hypothesis of equal means.

### *Clinical Improvement*

Ankle stability was observed at 1 and 2 years after surgery in all the patients. The negative results were recorded in the ankle varus stress tests and ankle anterior drawer tests. All the patients regained normal activities and normal gait during the 2‐year follow‐up duration, and no recurrence of CLAI or revision surgery was recorded.

### *VAS* *for Pain*

Decrease of VAS was recorded in both groups during the follow‐up period. The VAS in the two groups were similar at the 1‐ and 2‐year follow‐up (Table 3). Subgroup analysis showed that the patients in the horizontal mattress suture group revealed similar VAS scores at 1 and 2 years after the surgery (*t* = 1.624, *P* = 0.110). The VAS score of the free‐edge suture group at 2 years after surgery was significantly lower than that at 1 year after surgery (*t* = 2.280, *P* = 0.026). The absolute value of difference was a score of 0.48.

### *AOFAS* *Score*

Improvements of AOFAS were observed in both groups during the follow‐up period. The results in the two groups were comparable at the 1‐ and 2‐year follow‐ups (Table 3). The patients in the horizontal mattress suture group reported similar scores at 1 and 2 years after the surgery (*t* = −1.515, *P* = 0.135). The patients in the free‐edge suture group resulted in similar AOFAS at 1 and 2 years after the surgery (*t* = −1.411, *P* = 0.162).

### *KAFS* *Score*

Improvements of KAFS were observed in both groups during the follow‐up period. Patients of the free‐edge suture group achieved better KAFS 1 year (*t* = −2.879, *P* = 0.022, power = 0.809, absolute value = 4.65 score) and 2 years (*t* = 3.048, *P* = 0.003, power = 0.850, absolute value = 4.28 score) after the surgery compared with those of the horizontal mattress suture group (Table 3). The KAFS scores of the horizontal mattress suture group and the free‐edge suture group at 2 years after surgery were significantly higher than that at 1 year after surgery (*t* = −2.232, *P* = 0.029, absolute value = 3.75 score; *t* = −2.549, *P* = 0.013, absolute value = 3.38 score), respectively.

### *ATT* *Improvement*

The postoperative ATT of the horizontal mattress suture group and the free‐edge suture group was 3.81 ± 1.57 mm *vs* 3.93 ± 1.63 mm (*t* = 0.309, *P* = 0.759, power = 0.061), 3.34 ± 1.22 mm *vs* 3.40 ± 1.45 mm (*t* = 0.185, *P* = 0.854, power = 0.054) at 1 and 2 years after surgery, respectively. The differences were not statistically significant at either follow‐up points (Table 3). The patients in the horizontal mattress suture group and free‐edge suture group revealed similar ATT outcomes at 1 and 2 years after the surgery (*t* = 1.316, *P* = 0.193; *t* = 1.478, *P* = 0.144), respectively.

### *AJPS* *Improvement*

The AJPS in horizontal mattress suture and free‐edge suture groups showed no significant differences at 1 and 2 years after surgery (Table 4). The two suture fashion procedures resulted in similar AJPS outcomes at 1 and 2 years after the surgery (all *P* < 0.05).

**TABLE 4:** Comparison of postoperative ankle proprioception between the two groups at 1 and 2 years after surgery

Active joint position sense (degree)	Horizontal mattress suture group (n = 31)	Free‐edge suture group (n = 37)	*P* * value	Power^‡^
1 year				
Inversion 10°	7.57 ± 1.74	7.62 ± 1.90	0.910^†^	0.051
Inversion 20°	17.24 ± 2.81	17.43 ± 3.12	0.793^†^	0.058
Plantar flexion 10°	7.29 ± 1.83	7.48 ± 2.11	0.692^†^	0.068
Plantar flexion 20°	17.89 ± 2.55	18.16 ± 2.76	0.677^†^	0.070
2 years				
Inversion 10°	8.11 ± 1.36	8.08 ± 1.17	0.924^†^	0.051
Inversion 20°	18.33 ± 1.96	18.45 ± 1.88	0.799^†^	0.057
Plantar flexion 10°	7.72 ± 1.50	7.77 ± 1.64	0.896^†^	0.052
Plantar flexion 20°	18.87 ± 2.03	19.08 ± 1.82	0.658^†^	0.072

* A value *P* < 0.05 was set as statistically significant

^†^ *t* test

^‡^ Power is computed to reject the null hypothesis of equal means.

### *Evaluation in Activity Level*

The mean time of the return to full activity for patients in the horizontal mattress suture group was 10.38 ± 2.02 (range, 8 to 12) weeks *vs* 8.63 ± 2.31 (range, 8 to 12) weeks for those in the free‐edge suture group (*t* = 3.332, *P* = 0.001, power = 0.907). At the final follow‐up in the horizontal mattress suture group, 21 patients resumed pre‐injury sports activities and 10 patients chose leisure sports activities (non‐intense exercise) because of fear of secondary injury to the surgery site; in the free‐edge suture group, 25 people returned to pre‐injury sports activities and 12 chose leisure sports activities for the same reason. The exercise participation rates were similar between groups (*X^2^* = 0.001, *P* = 0.988).

### *Power and Subgroup Analysis*

Group sample sizes of 31 and 37 achieve less than 20.00% power (VAS, AOFAS, ATT, and AJPS, respectively) to reject the null hypothesis of equal means, with a significance level (alpha) of 0.050 using a two‐sided two‐sample unequal‐variance *t*‐test. Subgroup analysis (Table 5) showed that male patients in the free‐edge suture group observed better KAFS at 1 and 2 years after surgery than that in the horizontal mattress suture group (*t* = 2.826, *P* = 0.007, absolute value = 5.58 score; *t* = 2.445, *P* = 0.019, absolute value = 4.08 score), respectively. The female patients experienced higher KAFS score in the free‐edge suture group compared to the horizontal mattress suture group (*t* = 2.181, *P* = 0.039, absolute value = 4.68 score) at 2 years after surgery. Patients aged less than 30 years obtained higher KAFS score in the free‐edge suture group at 1 and 2 years after surgery (*t* = 2.241, *P* = 0.031, absolute value = 4.61 score; *t* = 2.176, *P* = 0.035, absolute value = 3.78 score), respectively. KAFS score at 2 years after surgery showed higher improvement with patients aged over 30 years in the free‐edge suture group compared to the horizontal mattress suture group (*t* = 2.278, *P* = 0.032, absolute value = 5.39 score). For patients with BMI lower 25.0 kg/m^2^, KAFS scores in the free‐edge suture group were better than that in the horizontal mattress suture group at 1 and 2 years after surgery (*t* = 3.033, *P* = 0.004, absolute value = 6.09 score; *t* = 3.288, *P* = 0.002, absolute value = 5.61 score), respectively. Patients with disease duration over 12 months revealed better KAFS scores in the free‐edge suture group than in the horizontal mattress suture group at 1 and 2 years after surgery (*t* = 2.119, *P* = 0.041, absolute value = 4.61 score; *t* = 2.339, *P* = 0.025, absolute value = 4.44 score), respectively. Similar ATT and AOFAS scores in both groups were recorded after the subgroup analysis of the age, gender, BMI, and disease duration (Table 5), respectively. A negative correlation (nonlinear relationship) was found between KAFS and VAS (Spearman correlation coefficient, −0.041; *P* = 0.658), and KAFS and age (Spearman correlation coefficient, −0.025; *P* = 0.787). A negative correlation (linear relationship) was found between KAFS and BMI (Spearman correlation coefficient, −0.330; *P* = 0.000). Thus, a higher score of KAFS will result if the VAS, age, or BMI is smaller.

**TABLE 5:** Subgroup comparison of postoperative clinical outcomes between the two groups

Subgroup	Variable		Horizontal mattress suture group (n = 31)	Free‐edge suture group (n = 37)	*P* * ^†^ value
Male	Patients		19	23	
	ATT	1 year	3.86 ± 1.56	3.96 ± 1.71	0.846
		2 years	3.42 ± 1.31	3.46 ± 1.43	0.926
	KAFS	1 year	80.56 ± 6.67	86.14 ± 6.11	0.007
		2 years	84.84 ± 5.94	88.92 ± 4.88	0.019
	AOFAS	1 year	89.12 ± 8.33	90.54 ± 9.27	0.608
		2 years	92.32 ± 5.92	92.87 ± 6.37	0.775
Female	Patients		12	14	
	ATT	1 year	3.73 ± 1.65	3.87 ± 1.55	0.826
		2 years	3.21 ± 1.12	3.30 ± 1.52	0.867
	KAFS	1 year	85.61 ± 6.68	88.84 ± 6.03	0.207
		2 years	88.52 ± 6.12	93.20 ± 4.82	0.039
	AOFAS	1 year	92.33 ± 8.81	92.39 ± 9.32	0.987
		2 years	94.52 ± 5.68	95.46 ± 6.51	0.701
Age, <30 y	Patients		18	25	
	ATT	1 year	3.91 ± 1.57	3.95 ± 1.69	0.938
		2 years	3.45 ± 1.31	3.57 ± 1.52	0.788
	KAFS	1 year	82.51 ± 7.15	87.12 ± 6.28	0.031
		2 years	86.14 ± 6.26	89.92 ± 5.12	0.035
	AOFAS	1 year	91.06 ± 8.56	92.15 ± 9.45	0.700
		2 years	94.68 ± 5.80	94.42 ± 6.33	0.891
Age, ≥30 y	Patients		13	12	
	ATT	1 year	3.69 ± 1.63	3.88 ± 1.58	0.770
		2 years	3.19 ± 1.12	3.06 ± 1.30	0.791
	KAFS	1 year	82.52 ± 7.12	87.23 ± 6.12	0.091
		2 years	86.43 ± 6.31	91.82 ± 5.44	0.032
	AOFAS	1 year	89.40 ± 8.71	89.35 ± 8.74	0.989
		2 years	91.09 ± 5.41	92.67 ± 6.85	0.527
Body Mass Index, <25 kg/m^2^	Patients		23	24	
	ATT	1 year	3.78 ± 1.61	3.85 ± 1.74	0.887
		2 years	3.22 ± 1.20	3.23 ± 1.44	0.980
	KAFS	1 year	82.95 ± 7.22	89.04 ± 6.54	0.004
		2 years	86.73 ± 6.29	92.34 ± 5.39	0.002
	AOFAS	1 year	91.83 ± 8.25	93.34 ± 9.59	0.566
		2 years	94.21 ± 5.58	95.34 ± 6.33	0.520
Body Mass Index, ≥25 kg/m^2^	Patients		8	13	
	ATT	1 year	3.91 ± 1.53	3.98 ± 1.63	0.923
		2 years	3.67 ± 1.31	3.51 ± 1.51	0.807
	KAFS	1 year	81.26 ± 6.71	86.01 ± 5.84	0.103
		2 years	84.91 ± 6.01	89.43 ± 5.02	0.078
	AOFAS	1 year	86.12 ± 8.33	89.94 ± 9.09	0.347
		2 years	90.17 ± 5.83	92.94 ± 6.62	0.343
Disease duration, <12 months	Patients		14	17	
	ATT	1 year	3.81 ± 1.52	3.94 ± 1.62	0.821
		2 years	3.34 ± 1.22	3.41 ± 1.54	0.891
	KAFS	1 year	82.48 ± 7.18	87.19 ± 6.34	0.062
		2 years	86.13 ± 6.32	90.23 ± 5.27	0.058
	AOFAS	1 year	90.32 ± 8.65	91.16 ± 9.36	0.799
		2 years	93.24 ± 5.90	94.12 ± 6.43	0.697
Disease duration, ≥12 months	Patients		17	20	
	ATT	1 year	3.82 ± 1.66	3.93 ± 1.68	0.843
		2 years	3.34 ± 1.25	3.40 ± 1.41	0.893
	KAFS	1 year	82.53 ± 7.11	87.14 ± 6.13	0.041
		2 years	86.37 ± 6.24	90.81 ± 5.31	0.025
	AOFAS	1 year	90.40 ± 8.67	91.31 ± 9.30	0.762
		2 years	93.12 ± 5.96	93.62 ± 6.65	0.813

AOFAS, American Orthopedic Foot and Ankle Society; ATT, Anterior Talar Translation; KAFS, Karlsson Ankle Functional Score.

* A value *P* < 0.05 was set as statistically significant

^†^ t test.

### *Complications*

In both groups, incisions were healed by first intention, and complications such as infection, implant reactions, tendon injury, and nervous or vascular injuries were not observed. During the 2‐years follow up duration, there was no implant rejection or suture rejection. No signs of suture anchor fixation failure or evidence of detachment was recorded. At the 2‐year follow‐up, all patients regained normal activities and ankle stability, and no recurrence of CLAI or revision surgery was recorded.

## Discussion

### *Key Finding of the Study*

The key finding of the current study was that all‐inside arthroscopic Broström–Gould surgery for CLAI with free‐edge suture provides better functional results compared with horizontal mattress suture. There were no differences in VAS, AOFAS, ATT, AJPS, or the rate of return to pre‐injury sports between the two suture groups at the 2‐year follow‐up. However, the KAFS is significantly higher and the recovery time is significantly shorter with free‐edge suture.

### *Current Literature About Suture Fashion of* *Broström–Gould* *Surgery*

Nery *et al*.^18^ retrospectively analyzed 38 consecutive cases of CLAI treated using all‐inside arthroscopic Broström–Gould surgery with horizontal mattress suture. After an average follow‐up of 9.8 years, the AOFAS improved to 90 scores (range, 44–100). The postoperative AOFAS scores were graded as excellent in 20 patients and good in 16 patients. The lateral ankle stability was obtained in all the patients without CLAI recurrence. Behrens and coworkers^19^ performed the Broström–Gould surgery with horizontal mattress suture fashion on 10 fresh cadaveric specimens. The lateral ankle stability was tested by using a Telos ankle stress apparatus with a 170 N load. No significant differences in the anterior drawer test or talar tilt test between the intact and Broström–Gould repaired state were measured. Xu and colleagues^20^ treated 28 CLAI patients with the Broström–Gould procedure and horizontal mattress suture. At the 2‐year follow‐up, the AOFAS score increased from 67.3 to 96.3 and the Foot and Ankle Ability Measure increased from 58.9 to 90.5. However, the suture strength of the horizontal mattress suture remains controversial. At final follow up, one patient had mechanical instability and underwent a revision operation. Yeo and colleagues^21^ retrospectively analyzed and compared outcomes of 99 CLAI cases (laxity group: 24 ankles; no laxity group: 75 ankles) treated with all‐inside Broström–Gould procedure utilizing free‐edge suture. At 1‐year follow‐up, both groups had achieved successful clinical and radiological outcomes. The all‐inside Broström–Gould procedure with free‐edge suture should be considered a reasonable method for CLAI patients regardless of generalized ligamentous laxity. Cottom *et al*.^22^ performed open Broström–Gould procedures with two suture anchors in free‐edge suture fashion on 12 fresh cadaveric specimens. The maximum load to failure reached 156.43 (range 83.69 to 192.00) N. Kim and coworkers^23^ examined 99 CLAI patients treated with the all‐inside arthroscopic Broström–Gould procedure; at 12 months after surgery, they found that AOFAS increased from 65.0 to 87.0, and the talar tilt decreased from 7.3º to 3.2º.

### *Clinical Outcome of All‐Inside Arthroscopic* *Broström–Gould* *Surgery with Horizontal Mattress Suture or Free‐Edge Suture*

The present study demonstrated that the all‐inside arthroscopic Broström–Gould surgery with horizontal mattress suture or free‐edge suture significantly improved functional results of VAS, AOFAS, KAFS, and ATT. This finding agrees with the conclusions of the previously mentioned investigations. We used the VAS, AOFAS, KAFS, and ATT to assess ankle stability and function in order to compare the results in both groups. The VAS scoring system focuses on the subjective evaluation of ankle pain. AOFAS is used for evaluating the pain, function, and alignment of the ankle^24^. KAFS is an important parameter for evaluating the stability and function of the ankle joint^25^, ^26^. ATT is the normally used ankle stability assessment index for CLAI^27^, ^28^. Proprioceptive functional rehabilitation after chronic ankle injury is important for ankle function recovery and patient satisfaction. We used the AJPS to assess ankle proprioceptive recovery in both groups. The AJPS is the most commonly used index for proprioceptive recovery evaluation^29^, ^30^. The postoperative AJPS between the two groups was comparable at the 1‐ and 2‐year follow‐ups.

The time of return to full activity and the rate of return to pre‐injury sports after CLAI repair are very important indicators of the benefit of the surgery^31^, ^32^. Regaining pre‐injury levels of physical activity is essential for functional recovery and self‐confidence building. Lateral ankle stability is associated with lateral ligament strength; a strong ligament allows patients to engage in postoperative rehabilitation immediately and confidently^33^. Based on the results of the present study, all‐inside arthroscopic Broström–Gould surgery for CLAI with free‐edge suture provided better ankle function compared with horizontal mattress suture.

### *Limitations of the Study*

This study has some limitations. First, we did not compare the biomechanical characteristics of horizontal mattress suture and free‐edge suture in the arthroscopic Broström–Gould procedures. Second, we only used the AJPS to assess proprioceptive function; this measurement method is simple and might not comprehensively assess all aspects of proprioception. Third, the follow‐up time was adequate at 2 years; however, we do not know whether the results will remain stable over time. Another limitation was that this was a retrospective study, creating the possibility of selection bias. Well‐designed prospective comparative studies and biomechanical analysis studies are needed to further confirm the long‐term functional outcomes and biomechanical characteristics of these procedures.

### *Conclusions*

Compared with the horizontal mattress suture technique, the all‐inside arthroscopic Broström–Gould procedure with free‐edge suture showed better short‐term advantages in terms of functional outcomes. KAFS is significantly higher and the recovery time is significantly shorter with free‐edge suture. However, patients did not subjectively notice a difference between free‐edge suture and horizontal mattress suture. We cannot recommend one procedure over the other, and we suggest that surgeons should perform the procedure with which they feel most comfortable.
